# KBWS: an EMBOSS associated package for accessing bioinformatics web services

**DOI:** 10.1186/1751-0473-6-8

**Published:** 2011-04-29

**Authors:** Kazuki Oshita, Kazuharu Arakawa, Masaru Tomita

**Affiliations:** 1 1Institute for Advanced Biosciences, Keio University, Fujisawa, 252-8520, Japan

## Abstract

The availability of bioinformatics web-based services is rapidly proliferating, for their interoperability and ease of use. The next challenge is in the integration of these services in the form of workflows, and several projects are already underway, standardizing the syntax, semantics, and user interfaces. In order to deploy the advantages of web services with locally installed tools, here we describe a collection of proxy client tools for 42 major bioinformatics web services in the form of European Molecular Biology Open Software Suite (EMBOSS) UNIX command-line tools. EMBOSS provides sophisticated means for discoverability and interoperability for hundreds of tools, and our package, named the Keio Bioinformatics Web Service (KBWS), adds functionalities of local and multiple alignment of sequences, phylogenetic analyses, and prediction of cellular localization of proteins and RNA secondary structures. This software implemented in C is available under GPL from http://www.g-language.org/kbws/ and GitHub repository http://github.com/cory-ko/KBWS. Users can utilize the SOAP services implemented in Perl directly via WSDL file at http://soap.g-language.org/kbws.wsdl (RPC Encoded) and http://soap.g-language.org/kbws_dl.wsdl (Document/literal).

## Background

With more than 1700 services listed in the BioCatalogue at the time of this writing [1], a significant number of biological resources and tools is provided as web-based services, in light of their advantages in interoperability, ease of use, and the lack of requirements for the compute infrastructure as well as the effort in installation and maintenance [2]. Several efforts have already utilized hundreds of these resources with the aim to effectively integrate them into a bioinformatics cyberinfrastructure, allowing the creation and management of research workflows through syntactic and semantic integration [3]. BioMoby project [4] maintains a repository of service descriptions with controlled vocabularies so that services can be discovered from their purpose and from their input/output data types, and MOWServ project [5] further classifies and curates the services in order to allow automatic workflow creation [6]. A number of clients with rich graphical user interface such as Seahawk [7] and Taverna [8] are also available as front-end research environment.

While the advent of web-based services is expected to proliferate further especially in light of the introduction of next-generation sequencers and their huge masses of data [9], local tools will continue to be important for numerous purposes, and web-based services need to be integrated with these local tools. Therefore, here we present the Keio Bioinformatics Web Service (KBWS), a collection of proxy client tools for 42 major bioinformatics web-based services in the form of European Molecular Biology Open Software Suite (EMBOSS) [10] associated software (EMBASSY) package of UNIX command-line utilities. EMBOSS is one of the most widely used collection of more than 200 bioinformatics tools, which takes advantage of the UNIX environment for "piping" with any other UNIX commands. With its Ajax Command Definitions (ACD), EMBOSS is well curated with controlled vocabularies and semantics so that the tools are highly documented (with *tfm *command), discoverable (with *wossname *command), and interoperable. Advantages of the EMBOSS platform also includes a unified sequence data retrieval system with Universal Sequence Address (USA) as well as the availability of many front-end user interfaces, such as JEMBOSS [11], SoapLab [12], wEMBOSS [13], and EMBOSS Explorer [14].

## Implementation

KBWS is composed of two parts: a proxy web server providing SOAP service wrapper to bioinformatics web services, and UNIX command-line clients in the form of EMBOSS tools that access the proxy web server. By making the clients access the proxy server instead of the original service providers, the clients can be lightweight. Therefore, same client tool can be used without update or maintenance even the original service providers change their formats, and KBWS can utilize bioinformatics web-based services provided in any protocol (such as SOAP, REST - Representational State Transfer, or browser-based CGI - Common Gateway Interface) of any versions [15]. This proxy server is able to deal immediately with specification change of original service by regular automatic monitoring to check whether the proxy server returns correct report. Even if the original server is down, KBWS is able to stably provide same services without update to the client tools, by switching the server to analogous web-based services at the proxy server. Moreover, users can access the proxy server via SOAP, which provides all 42 services under the same programming interface defined in a single Web Service Description Language (WSDL). WSDL files that are provided as RPC Encoded style and Document/literal style allow access to KBWS from web-based services client software or programming languages. The proxy server provides access to 9 SOAP services, 3 REST services, 31 CGI services and 1 service installed in the web server, and is implemented using SOAP::Transport::HTTP Perl module. Three services provided by NCBI, EBI, and DDBJ, respectively, are used for a single tool *kblast*, and therefore there is a total of 42 tools accessing 44 services. List of supported web services in KBWS is shown in Table 1.

**Table 1:** List of supported services

Category	Service Name	References	Tool Name
***ALIGNMENT LOCAL***	BLAST	Altschul *et al.*, 1990	kblast
		McWilliam et al., 2009	
	SSEARCH	Mackey *et al.*, 2002	kssearch
		McWilliam et al., 2009	

***ALIGNMENT MULTIPLE***	ClustalW	Larkin *et al.*, 2007	kclustalw
		McWilliam et al., 2009	
	MAFFT	Katoh *et al.*, 2009	kmafft
	Kalign	Lassmann *et al.*, 2006	kkalign
		McWilliam et al., 2009	
	MUSCLE	Edgar, 2004	kmuscle
	T-Coffee	Notredame *et al.*, 2000	ktcoffee
		McWilliam et al., 2009	

***NUCLEIC COMPOSITION***	WebLogo	Crooks *et al.*, 2004	kweblogo

***NUCLEIC GENE FINDING***	GeneMarkHMM	Lukashin *et al.*, 1998	kgenemarkhmm
	GLIMMER	Delcher *et al.*, 1999	kglimmer
	tRNAscan-SE	Lowe *et al.*, 1997	ktrnascan_se

***PROTEIN LOCALIZATION***	PSORT	Nakai *et al.*, 1991	kpsort
	PSORT2	Nakai *et al.*, 1991	kpsort2
	PSORT-B	Yu *et al.*, 2010	kpsortb
	WoLF PSORT	Horton *et al.*, 2007	kwolfpsort

***PROTEIN MOTIFS***	Phobius	Lukas *et al.*, 2004	kphobius
		McWilliam et al., 2009	

***PROTEIN PROFILES***	dbFetch	Labarga *et al.*, 2007	kfetchdata, kfetchbatch
		McWilliam et al., 2009	

***RNA 2D STRUCTURE DISPLAY***	Centroid Fold	Sato *et al.*, 2009	kcentroidfold
	RNAfold	Hofacker *et al.*, 1994	krnafold

***PATHWAY MAPPING***	PathwayProjector	Kono *et al.*, 2009	kpathwayprojector

***PHYLIP Tools***	PHYLIP	Lim *et al.*, 1999	kclique, kcontml
			kdnacomp, kdnadist
			kdnainvar, kdnaml
			kdnamlk, kdnapars
			kdnapenny, kdollop
			kdolpenny, kfitch
			kgendist, kkitsch, kmix
			kneighbor, kpenny
			kprotdist, kprotpars
			krestml, kseqboot

The software categories are based on those used in EMBOSS. Complete listings including full references and URLs are available at our website (http://www.g-language.org/kbws/).

Each of the 42 UNIX command-line clients accesses a unique bioinformatics web service through KBWS proxy. These tools are implemented as EMBASSY package in C programming language with gSOAP Toolkit [16], and are available under GNU General Public License from http://www.g-language.org/kbws/ and GitHub repository http://github.com/cory-ko/KBWS. Detailed documentation for each of the tools are available through the EMBOSS *tfm *(The Fine Manual) command, and tools can be searched and discovered with *wossname *utility. As an EMBOSS package, KBWS can be utilized with graphical user interfaces through JEMBOSS and wEMBOSS, and browser-based access with EMBOSS Explorer is also available at our website (http://soap.g-language.org/kbws/emboss_explorer/).

## Results and Discussion

**Figure 1:**
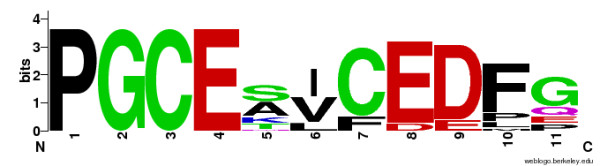
**Sequence Logo created by example workflow**. This sequence logo image is created by example workflow for generating a sequence logo image for a set of amino acid sequences of FOXP2. This workflow includes KBWS Tools (*kblast, kmuscle, kweblogo*), EMBOSS tool (*extractalign*), and other local tools (*sed, uniq*).

The following set of commands comprises a workflow for generating a sequence logo image for a set of amino acid sequences of FOXP2 [17], using BLAST web service (*kblast*), extracting the list of IDs (*sed *and *uniq*), aligning the sequences with MUSCLE (*kmuscle*), extracting a certain region from the alignment (*extractalign*), and generating its sequence logo (*kweblogo*). Output from this workflow is shown in Figure 1. The definition of "swissprot" database used in the following workflow is available at http://soap.g-language.org/kbws/embossrc.

# search similar sequence in Swiss-Prot database using BLAST

% kblast swissprot:FOXP2_HUMAN -d swissprot -format k1 -eval 1e-50 -outfile kblast.out

# extract ID list from BLAST report

% sed 's/^\(.*\)\.[1-9]/swissprot:\1/g' kblast.out | uniq > match_list.out

# multiple sequence alignment using MUSCLE

% kmuscle @match_list.out -outfile kmuscle.fasta

# Extract a region from the alignment

% extractalign -regions '420-430' kmuscle.fasta -outseq extractalign.fasta

# Generation of sequence logos using WebLogo

% kweblogo extractalign.fasta

Other example workflows are available at myExperiment (http://www.myexperiment.org/) website as Taverna workflow (http://www.myexperiment.org/users/1938/workflows).

Sample codes written by Perl, Ruby, Python and Java are also available at http://www.g-language.org/wiki/kbws/.

KBWS provides 42 web-based services as an EMBOSS package. EMBOSS is already widely used in numerous laboratories, and therefore users can readily integrate web-based services with their EMBOSS tools and UNIX commands using KBWS. Moreover, KBWS adds the advantages of web-based services to the many functionalities of EMBOSS, especially for tools like BLAST search that require large regularly updated databases that are difficult to be installed and maintained with EMBOSS in traditional manner. KBWS also does not require any external software or data for installation except for EMBOSS. As an EMBOSS package, KBWS is well-documented and can take advantage of the documentations and discovery tools, and can be used from rich clients such as JEMBOSS, wEMBOSS, and EMBOSS Explorer.

## List of abbreviations

ACD: Ajax Command Definitions; CGI: Common Gateway Interface; EMBOSS: European Molecular Biology Open Software Suite; KBWS: Keio Bioinformatics Web Service; REST: Representational State Transfer; USA: Universal Sequence Address; WSDL: Web Service Description Language.

## Competing interests

The authors declare that they have no competing interests.

## Authors' contributions

KO developed the software, KA conceived of the project, and KO and KA designed the software and drafted the manuscript. MT supervised the project. All authors have read and approved the final manuscript.
